# Report of intraosseous intravascular papillary endothelial hyperplasia associated with an odontogenic cyst in the maxilla and literature review

**DOI:** 10.1186/s13000-024-01505-1

**Published:** 2024-06-12

**Authors:** Mateus José Dutra, Ana Lia Anbinder, Christyan Moretti Pereira, Beatriz Afonso Chiliti, André Caroli Rocha, Estela Kaminagakura

**Affiliations:** 1 Institute of Science and Technology, Department of Biosciences and Oral Diagnosis, São Paulo State University (UNESP), São José dos Campos Av. Engenheiro Francisco José Longo Avenue, 777/778, Jardim São Dimas, São José dos Campos, SP 12245000 Brazil; 2 Oral and Maxillofacial Surgery and Traumatology Service, Hospital das Clínicas of the University of São Paulo Medical School, São Paulo, Brazil

**Keywords:** Odontogenic cysts, Oral pathology, Mouth neoplasms, Blood vessels, Hyperplasia

## Abstract

Intravascular papillary endothelial hyperplasia (IPEH) represents an uncommon reactive endothelial hyperplastic proliferation. A 46-year-old man experienced increased volume in the right maxilla, elevation of the nasal ala, and swelling of the hard palate with a reddish hue for 3 months. Computed tomography revealed an expansive hypodense region and cortical bone destruction associated with an impacted supernumerary tooth and an endodontically treated tooth. Under the differential diagnoses of a radicular cyst, dentigerous cyst, and ameloblastoma, an exploratory aspiration and incisional biopsy were performed. This revealed the formation of blood vessels of various diameters lined by endothelium, forming intravascular papillae positive for CD-34. The definitive diagnosis was IPEH, and the patient was treated by embolization and surgery. Histological analysis confirmed the presence of IPEH associated with an odontogenic cyst. After 12 months of follow-up, no recurrence was observed. Also, we reviewed case reports of IPEH affecting the maxilla and mandible. Fourteen intraosseous cases were reported in the maxilla and mandible, with a preference for males and affecting a wide age range. Complete surgical excision was the treatment of choice, and recurrences were not reported. The pathogenesis of IPEH is controversial and may originate from trauma or inflammatory processes. To the best of our knowledge, this is the first report of an association of IPEH with an odontogenic cyst. The importance of IPEH in the differential diagnosis of intraosseous lesions in the jaws is emphasized, and preoperative semiotic maneuvers are needed to prevent surgical complications.

## Introduction

Intravascular papillary endothelial hyperplasia (IPEH) was first described by Pierre Masson in 1923 and is currently considered a reactive endothelial hyperplastic proliferation [1, 2]. It is uncommon in the maxillofacial region but, when it occurs, tends to affect soft tissues [3].

Although IPEH has been described as a reactive hyperplastic proliferation [1, 2], little is known about its pathogenesis. Its etiological formation can be traumatic or inflammatory [4], because of endothelial cell proliferation resulting from a thrombus or secondary to vascular lesions such as varices, hemangiomas, and pyogenic granulomas [5]. The management of IPEH is generally through surgical excision, and the recurrence rate is low [6].

IPEH rarely affects the jaw bones. However, it can reach significant sizes and, due to its vascular nature, can bleed profusely, posing a life-threatening risk. In this scenario, it is important for clinicians and pathologists to be aware of this uncommon lesion and to include it in their differential diagnosis. We report, to the best of our knowledge, a previously unreported association between an odontogenic cyst and IPEH, and review the published cases in the English literature, which may facilitate the diagnosis of similar lesions in the future.

## Case report

A 46-year-old man sought help from the Oral Diagnosis Service of the Institute of Science and Technology (São Paulo State University/UNESP), complaining of a volume increase in his right maxilla over the previous 3 months. His medical history included asthma, rhinitis, and being allergic to dipyrone, diclofenac, acetylsalicylic acid, penicillin, and cephalosporins. He mentioned having undergone septoplasty 7 years previously and tooth extractions in the region 2 years ago .

**Fig. 1:**
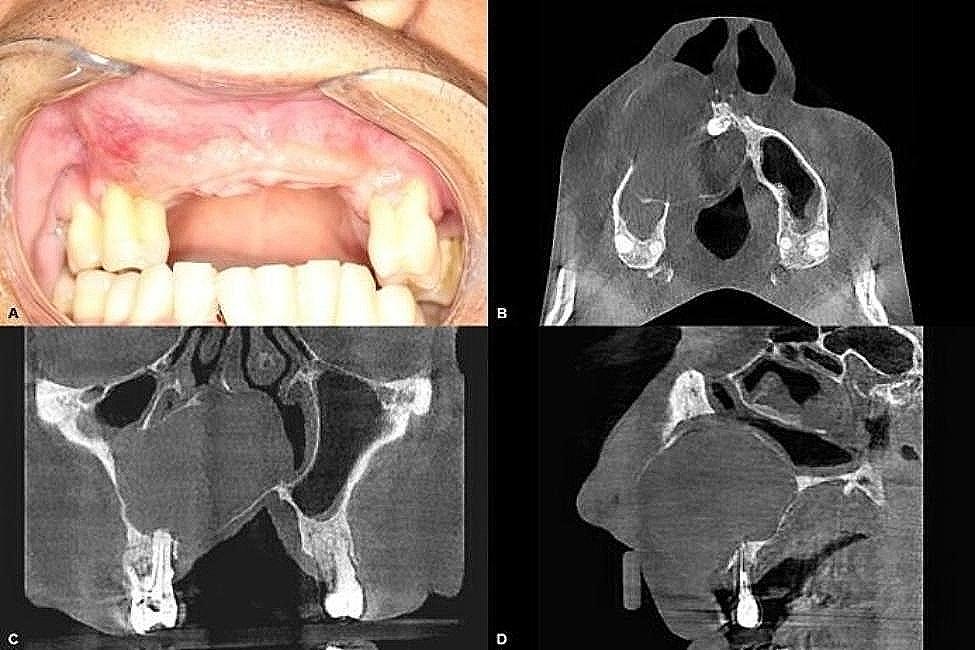
Initial clinical photograph and tomographic sections (**A**). Volume enlargement in the right maxilla. (**B**). Axial section showing association of the lesion with a supernumerary tooth. (**C**). Coronal section showing association of the lesion with the periapex of a tooth treated endodontically. (**D**). Sagittal section.

He reported that endodontic retreatment and periodontal scaling had been performed on his right maxillary first premolar but that his clinical condition had not improved. On extraoral physical examination, an increased size of the right maxilla with elevation of the nasal wing was noted. Intraorally, swelling in the region of the maxillary incisors to premolars on the same side, elevation of the hard palate similar in color to the adjacent mucosa with some reddish areas (telangiectatic), and no painful symptoms on soft palpation were noted (Fig. 1).

Tomographic examination revealed a mixed, hypodense area causing the expansion and resorption of the bone cortices, extending into the maxillary sinus and crossing the midline. The lesion was observed to be in close contact with a supernumerary tooth present in the midline region and was also associated with the apex of the right maxillary first premolar that had received endodontic treatment (Fig. 1).

Under the differential diagnoses of radicular cyst, dentigerous cyst, and ameloblastoma, an exploratory puncture was performed. This was positive for sanguineous content and was followed by an incisional biopsy that during surgery showed intense bleeding.

**Fig. 2:**
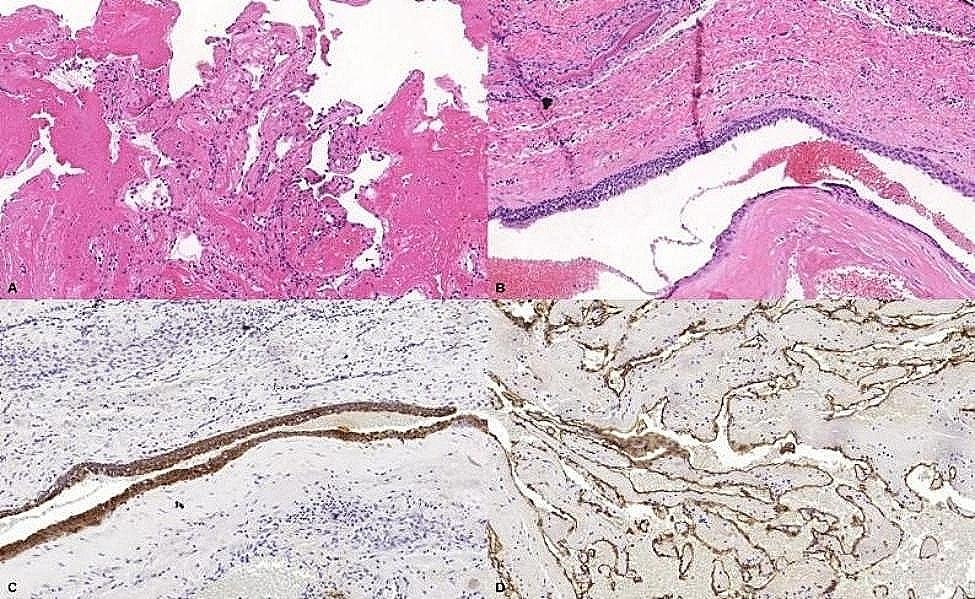
Histological sections of the incisional biopsy and immunohistochemical reactions. (**A**). Areas containing papillary projections lined by endothelium amidst fibrin (Hematoxylin and Eosin, original magnification 50×). (**B**). Areas of epithelium present in the lamina propria (Hematoxylin and Eosin, original magnification 50×). (**C**). Epithelium present in the lamina propria showing positivity for AE1/AE3 (original magnification 50×). (**D**). Connective tissue lined by endothelium with positivity for CD34 (original magnification 50×)

Histological sections of the incisional biopsy revealed mucosal fragments lined with stratified squamous epithelium. In the underlying connective tissue, areas of pseudostratified columnar or cuboidal epithelium with 2 cellular layers were observed and interpreted as possible cystic epithelium. In addition, an area of granulation tissue associated with congested blood vessels, and hemorrhagic areas occupied by fibrin and organizing thrombus were observed toward the depth of the sections. Blood vessels of various diameters lined by regular endothelium and with intravascular papillary formations were observed. Small nests and cords of cells with foamy cytoplasm interpreted as macrophages, and moderate lymphoplasmacytic inflammatory infiltrate were dispersed throughout the thrombotic areas of fibrin and hemorrhage (Fig. 2).

With the provisional diagnosis of a lesion with areas of intravascular endothelial proliferation, immunohistochemical reactions were performed for AE1/AE3, vimentin, and CD34. The epithelium was positive for AE1/AE3, while the endothelium was positive for vimentin and CD34 (Fig. 2). The diagnosis was a lesion with areas of intravascular endothelial proliferation, suggestive of IPEH. The patient was treated by embolization, followed by surgery.

Carotid angiography and embolization of the lesion were performed 24 h before surgical intervention. Upon initial evaluation, a heterogeneous tumor blush with moderately defined contours was observed in the anterior palatal region, predominantly on the right side. The lesion was mainly opacified by branches of the right internal maxillary artery (infraorbital, descending palatine), with lower opacification from the contralateral homonymous artery, facial artery, and right ascending palatine artery. A central area was found with low arterial density but with a dilated venous component and slight early enhancement. Subsequently, embolization was performed using PVA microspheres (Polyvinyl alcohol) of 150–255 μm and Gelfoam.

**Fig. 3:**
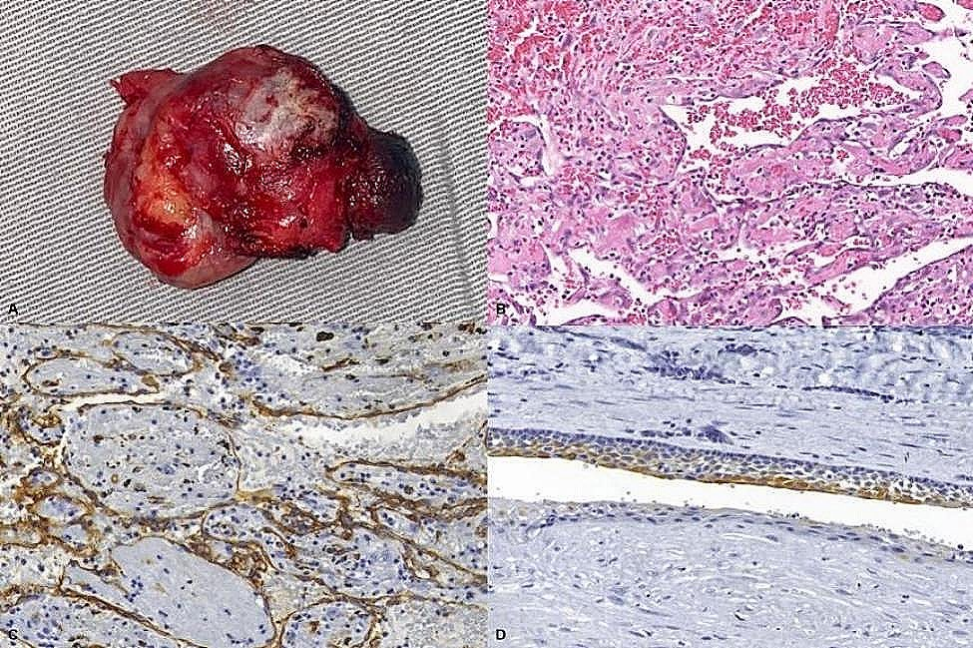
Surgical specimen and its histological sections and immunohistochemical reactions. (**A**). Macroscopic aspect of the surgical specimen. (**B**). Papillary projections lined by endothelium amidst hemorrhage (Hematoxylin and Eosin, original magnification 100×). (**C**). Connective tissue lined by endothelium with positivity for CD34 (original magnification 100×). (**D**). Epithelium of the odontogenic cyst associated with IPEH with positivity for CK-19 (original magnification 100×)

Later, the patient underwent surgical excision (Fig. 3). The material was sent for histopathological examination, confirming the diagnosis of IPEH associated with an odontogenic cyst and which was positive for CD34 in the papillary projections and for CK19 in the cystic epithelium (Fig. 3). The patient has been followed for 12 months without signs of recurrence.

## Literature review

The PubMed and SCOPUS databases were searched in March 2024 for reported cases of IPEH involving the jaws. The search terms used were “intravascular papillary endothelial hyperplasia AND maxilla AND Masson’s tumor” and “intravascular papillary endothelial hyperplasia AND mandible AND Masson’s tumor.” Both searches yielded 4 results. Of these 8 articles, an additional 7 reports and case series of IPEH affecting the jaws were retrieved from citations, totaling 15 articles to be included in the literature review. Inclusion criteria required articles to be reports and case series of IPEH affecting the intraosseous maxilla or mandible, published in English, with information on sex, age, anatomic site, signs and symptoms, imaging characteristics, histological features, treatment, and recurrence of the lesion. Exclusion criteria were diagnoses of IPEH not involving the maxilla and mandible, extraosseous locations, absence of 2 or more clinical data investigated in the inclusion criteria, and unavailability of the report for full reading. As a result, 3 articles were excluded, and 12 were included in the literature review. Data from reported cases included in the review and from this report (*n* = 15) are shown in Table 1.

**Table 1:** Clinical, imaging, histological, and treatment data of reported cases of intraosseous IPEH in the maxillary bones

Author	Gender	Age	Anatomic site	Signs and Symptoms	Imaging Characteristics	Histological Characteristics	Treatment
Komori et al., 1984 [12]	F	49	Mandible	None	Multilocular radiolucency	Papillary structures covered by flat or swollen endothelial cells	Surgical removal
Stern et al.,1991 [19]	F	17	Maxillary Sinus	Headaches and swelling	Soft tissue density mass	Papillary endothelium-lined spaces with fibrin and thrombus	Surgical removal
Lancaster et al., 1998 [20]	F	67	Maxillary Sinus	Nasal obstruction, rhinorrhea, and pain	Opacity in the left maxillary sinus without bone erosion	Small blood vessels with endothelium-lined structures	Endoscopic removal
Wang et al., 2009 [21]	M	42	Nasal Fossa, Maxillary, Ethmoidal, and Frontal Sinuses	Nasal obstruction, rhinorrhea, epistaxis, and headache	Soft tissue density mass	Papillary structures lined by a single layer of endothelial cells	Endoscopic removal
Xu and Li, 2014 [13]	M	14	Mandible	Facial swelling and trismus	Multilocular radiolucency with expansion and cortical perforation	Hemorrhagic areas with endothelium-lined structures positive for CD34	Surgical removal
Tanio et al., 2016 [14]	M	75	Mandible	Pain	Multilocular radiolucency with cortical destruction	Intravascular proliferations of papillae lined by endothelium positive for CD34	Surgical removal
D’Aguanno et al., 2019 [22]	F	67	Maxillary Sinus	Nasal obstruction, pain, and rhinorrhea	Opacity in the maxillary sinus with bone erosion	Dilated vessels with presence of organized thrombi	Open and endoscopic surgery
Mirmohammadsadeghi et al., 2019 [15]	M	45	Mandible	Fetid mouth mass, asymmetry, and bulging	Multilocular radiolucency with expansion, cortical destruction, root displacement, and resorption	Papillae covered by endothelial cells, with dilated vascular lumen with thrombosis and hemorrhage	Hemimandibulectomy with soft tissue resection
Cooke et al., 2020 [23]	M	28	Maxillary Sinus and Nasal Cavity	Epistaxis, headaches, and facial sinus pain	Mass in the maxillary sinus with erosion of multiple bony structures	Intravascular papillary projections lined by endothelial cells positive for CD31 and CD34	Endoscopic approach
Eguchi et al., 2020 [16]	M	55	Mandible	None	Multilocular radiolucency	Papillary fibrous tissue, lined by endothelium positive for CD34, negative for D2-40 and AE1/AE3, with low Ki-67	Surgical removal
Voruz et al., 2022 [24]	M	46	Maxillary Sinus	Nasal obstruction and bloody rhinorrhea	Soft tissue density mass	Areas of intravascular papillary endothelial hyperplasia in an organized hematoma	Transnasal resection
Voruz et al., 2022 [24]	M	76	Maxillary Sinus and Nasal Fossa	Epistaxis and rhinorrhea	Soft tissue density mass	Papillary endothelial hyperplasia within an organizing hematoma	Surgical removal
Voruz et al., 2022 [24]	F	33	Maxillary Sinus	Rhinorrhea, intraorbital pressure, and headache	Soft tissue density mass with surrounding bone integrity	Multiple fibrohemorrhagic changes compatible with Masson’s tumor	Endoscopic resection
Bofano et al., 2024 [18]	F	66	Mandible	None	Multilocular radiolucency with expansion and cortical resorption	Intravascular proliferations of papillary processes lined by endothelium positive for CD31 and CD34	Surgical removal
Present case	M	46	Maxilla	Swelling	Unilocular radiolucency with cortical resorption, associated with an impacted tooth	Intravascular papillae lined by endothelium positive for CD34 associated with an odontogenic cyst	Surgical removal

M: Male; F: Female

## Discussion

IPEH has been defined as a reactive vascular proliferation composed of endothelial cells alongside an organizing thrombus [6]. However, its pathogenesis remains uncertain, and it may occur after a traumatic or inflammatory event [4], through endothelial cell proliferation resulting from a thrombus or secondary to vascular lesions [5]. With thrombus formation, there is blood stasis, and macrophages can be activated, they stimulates endothelial cell proliferation with papillary formations by releasing fibroblast growth factor contributing to a continuous process that leads to its progression [3, 7, 8].

In our review of cases of intraosseous IPEH in the maxilla and mandible (*n* = 15), we found a slight tendency to affect the maxillary sinus (*n* = 9) compared with the mandible (*n* = 6), with a slight predilection observed in males (*n* = 9) over a wide age range (14 to 76 years). In the mandible, 50% of patients were asymptomatic, while in the maxillary sinus, 100% of patients complained of pain, obstruction, rhinorrhea, or swelling. In the mandible, imaging tests showed perforations and bone expansions in most cases (66.67%), while, in the maxillary sinus, this was not frequent (33.33%). The treatment of choice was complete surgical excision (100%), and, in many cases affecting the maxillary sinus, an endoscopic approach was performed, with no case showing recurrence (100%).

In this case report, the patient had a history of septoplasty, multiple dental extractions because of infections, endodontic treatment in the region, and an impacted supernumerary tooth associated with the lesion. We suggest that the presence of an impacted tooth or previous inflammatory/infectious processes may have given rise to an odontogenic cyst. Then, due to the inflammatory condition, IPEH developed within the cystic capsule.

The histological features of a radicular cyst after endodontic therapy, when the infectious stimulus is diminished, resemble those of an inflamed dentigerous cyst. The epithelial lining may consist of a few layers of nonkeratinizing squamous cells, and the interface between the epithelium and connective tissue can be flat, with a fibrous wall showing variable infiltration of inflammatory cells. In these cases, the clinical characteristics, and the location of the radiolucent area (around the apex or around the crown of an unerupted tooth) are key factors for the final diagnosis. In our case, the lesion involved the apical area of an endodontically treated tooth as well as the crown of a supernumerary tooth. Additionally, as IPEH developed within the cystic capsule causing compression of the cyst, a definitive classification was not possible. Although further classification of the cyst was challenging, the positive staining of the epithelium for CK19 indicates its odontogenic origin. Clinically, painless volume enlargement with mobility and areas of reddish discoloration is commonly observed in the extremities [9–11]. In the maxillofacial region, these clinical characteristics are seen in soft tissues. If intraosseous, this may lead to other pathologies such as cysts and odontogenic tumors , which were the main differential diagnoses in this case.

To our knowledge, this is the first case of an IPEH in the maxilla associated with an odontogenic cyst published in the English literature. Only seven cases of mandibular intraosseous IPEH have been reported [12–18], and also eight affecting the maxillary sinus [19–24], none have been described as being associated with odontogenic lesions.

In the present case, unlike other authors [12–16], we did not observe root resorption. However, areas of bone expansion, cortical resorption, maxillary sinus obliteration, and nasal septum destruction were noted. Several authors have also reported these characteristics [13, 15]. Since these are nonspecific characteristics, we suggest that the differential diagnosis of IPEH should be considered in patients presenting with radiolucencies affecting the jaws .

Histopathological criteria for the diagnosis of IPEH include the presence of a proliferation containing papillary projections connected to the endothelial surface [2, 9, 25]. Three histopathological subtypes have been described: arising in dilated vessels; arising within vascular lesions; and extravascular, arising from hematomas [26]. IPEH differs histopathologically from vascular lesions, particularly angiosarcoma, which is clinically aggressive and microscopically shows malignant cytological features such as endothelial layers of vascular spaces, atypical mitoses, necrosis, nuclear pleomorphism, and the invasion of adjacent tissues [2, 9, 11]. Conversely, IPEH does not show atypical cells and mitoses, necrosis, nuclear pleomorphism, and invasion of adjacent tissues [2, 9, 19, 23, 25]. However, we observed a broad lesion that destroyed important anatomic structures.

Some immunohistochemical reactions may contribute to the diagnosis of IPEH, such as CD34 and vimentin [16]. Ki-67 may also help in cases where there is doubt in differentiating between IPEH and angiosarcoma, as the latter generally exhibits a high expression of Ki-67 [27].

During the incisional biopsy, there was intense bleeding as the lesion may have been located intravascularly [26]. Thus, prior embolization may prove beneficial in mitigating bleeding during surgical excision [6, 14], as observed in this case report. In addition to surgery, chemotherapy, and radiotherapy have been described as adjuvant treatments for IPEH, in cases of multiple lesions or when complete resection is not possible [28]. Twelve months after embolization and surgery treatment, the patient showed no signs of recurrence at follow-up.

Although IPEH is currently classified as a reactive lesion, in some cases, it can grow rapidly and progressively, even in newborns, raising the question of whether it is not a neoplasm as it had been previously classified [29]. However, we emphasize that it can present intraosseously and be associated with other lesions in the maxillofacial region and should therefore be considered in the differential diagnosis. Clinical and imaging findings are nonspecific and may mimic other disorders; therefore, an exploratory puncture may indicate the diagnosis, which is generally confirmed by biopsy. The histopathological findings of routine staining confirm the diagnosis, which can be facilitated with immunohistochemistry. The treatment of choice is complete surgical excision, and recurrences may occur only if the lesion is not completely removed.

## Data Availability

No datasets were generated or analysed during the current study.
